# Mitochondrial Genomes of the Robberflies *Clephydroneura jiangxiensis* and *Maira xizangensis* (Diptera: Asilidae) and Phylogeny of Three Superfamilies

**DOI:** 10.3390/genes16050561

**Published:** 2025-05-08

**Authors:** Keyao Zhang, Junhui Lu, Sheng-Quan Xu

**Affiliations:** College of Life Sciences, Shaanxi Normal University, Xi’an 710119, China; keyaozh@163.com (K.Z.); lujunhui@snnu.edu.cn (J.L.)

**Keywords:** Asiloidea, mitochondrial genome, phylogeny

## Abstract

Background: Asilomorpha, an infraorder of predatory Diptera (Brachycera), is of significant evolutionary interest due to their remarkable ecological diversity, broad size range, and specialized feeding behaviors. However, phylogenetic studies of this group have been limited by sampling challenges. Methods: In this study, we sequenced the complete mitochondrial genomes of two Chinese endemic species, *Clephydroneura jiangxiensis* (*C. jiangxiensis*) and *Maira xizangensis* (*M. xizangensis*), using whole-genome random sequencing. By integrating these novel data with published sequences from NCBI, we reconstructed the phylogeny of three superfamilies (Asiloidea, Empidoidea, and Nemestrinoidea). Results: Both mitochondrial genomes exhibit the typical 37 genes (13 protein-coding genes, 22 tRNAs, and 2 rRNAs) and display pronounced AT bias. Congruent results from maximum likelihood analysis and Bayesian inference strongly supported the ideas that both new species are placed in Asilidae and that the Asilidae family is monophyletic. However, relationships among the three superfamilies remain unclear. Our results suggest that (1) although Asiloidea and Nemestrinidea are closely related, the potential positioning of Nemestrinoidea as an independent superfamily is worth investigating; and (2) Empidoidea may form a sister group to Asiloidea + Nemestrinidae, though this hypothesis requires further corroboration given the basal position of *Hemipenthes hebeiensis* (Bombyliidae). Conclusions: These findings highlight the need for expanded taxon sampling, particularly of underrepresented families, to resolve deep-level relationships within Asilomorpha. Clarifying the phylogenetic relationships within Asilomorpha will facilitate future investigations into their evolutionary origins and the evolution of characteristic traits.

## 1. Introduction

Asilomorpha, a diverse clade within Diptera Brachycera, exhibits a global distribution across varied ecosystems including forests, grasslands, agricultural systems, wetlands, and deserts [1]. Ecologically, most Asilomorpha species are recognized as predators, while certain members of this group additionally serve pollinator roles. Notably, Nemestrinidae possess elongated mouthparts adapted for pollen feeding, with fossil evidence suggesting that *Hirmoneura* may have evolved pollination behavior as early as the Mid-Cretaceous [2]. The genus *Protonemestrius*, characterized by an exceptionally elongated proboscis, represents one of the earliest known flower-visiting Diptera, demonstrating specialized morphological adaptations for nectarivory [3]. Larval feeding strategies further highlight ecological diversity [4]: the larvae of stiletto flies (Therevidae) are predators of fossorial arthropods, suggesting their potential as biocontrol agents. The larvae of bee flies (Bombyliidae) exhibit three distinct trophic modes: endoparasitism (e.g., *Anthrax fur* larvae developing within wasp pupae [5]), ectoparasitism (e.g., *Heterostylum robustum* larvae attached to adult Braconidae [6]), and egg-pod predation (e.g., *Systoechus somali* consuming desert locust eggs) [7].

Asilomorpha historically comprised three superfamilies: Asiloidea, Bombylioidea, and Acroceridae [8]. However, this infraorder has been rejected as a valid taxonomic group due to its demonstrable paraphyly, with Rohdendorf’s original concept being based solely on simplified morphological synapomorphies [9]. Phylogenetic relationships and taxonomic boundaries within this assemblage remain unresolved, with persistent conflicts between morphological and molecular datasets [10,11]. Conventional taxonomic approaches based on adult morphological characters and larval respiratory morphology show limited reliability for deep-level phylogenetic inference, owing to widespread homoplasy and insufficient phylogenetic signal retention [12]. The taxonomically unstable positions of key transitional groups—particularly *Hilarimorpha* and *Apystomyia*, which have been variably placed within both Bombylioidea and Asiloidea—exemplify the fundamental limitations of current systematic frameworks [11,13].

The superfamily Nemestrinoidea was originally proposed to comprise three components: the small families Nemestrinidae and Acroceridae, along with the species-rich Bombyliidae [14]. However, Yeates’ classification system placed Bombyliidae within Asiloidea based on several morphological characteristics, including inconsistent wing venation patterns and the parasitic nature of their larvae [15]. Molecular phylogenetic analyses have subsequently confirmed the monophyly of Nemestrinidae and Acroceridae while rejecting the inclusion of Bombyliidae within this clade [16]. The monophyly of the Asiloidea superfamily itself remains questionable, particularly in relation to Eremoneura. Nuclear marker studies (e.g., CAD and 28S rDNA) have challenged both the integrity of Asiloidea and the proposed sister-group relationship between Bombylioidea and Eremoneura [11,13]. Wiegmann’s work further suggested that Nemestrinoidea (Nemestrinidae + Acroceridae) may share a closer phylogenetic affinity with Asiloidea than previously recognized. Mitochondrial genomic data have revealed significant incongruences with morphological topologies, especially within Bombyliidae where tribal relationships conflict with traditional classification based on adult wing venation and larval characteristics [17]. Empidoidea represent the most consistently supported monophyletic lineage within Asilomorpha, though their familial composition remains debated [18]. Sinclair’s classification system recognized five constituent families (Empididae, Hybotidae, Atelestidae, Brachystomatidae, and Dolichopodidae) based on morphological synapomorphies in antennae, mouthparts, thoracic structures, wings, and terminalia [19]. In contrast, Grichanov proposed an epifamily Dolichopodoidae (=Dolichopodidae sensu lato) encompassing Dolichopodidae, Microphoridae, and Parathalassiinae [20]. These conflicting classification schemes underscore the ongoing uncertainty surrounding the higher-level systematics of Asilomorpha.

This study investigated the phylogenetic relationships among three dipteran superfamilies (Asiloidea, Empidoidea, and Nemestrinoidea) through comparative mitochondrial genomics. Given their structural simplicity and high sequence conservation, mitochondrial genomes were selected as the primary marker to establish the foundational phylogeny of Asilomorpha. Building upon prior molecular and morphological evidence, we sequenced and annotated the complete mitochondrial genomes of two Chinese endemic Asilidae species—*C. jiangxiensis* and *M. xizangensis*—and compiled 76 mitochondrial genomes (72 ingroup taxa + 4 outgroups) from NCBI. Nucleotide substitution analyses revealed significant heterogeneity in both: (1) saturation patterns across codon positions and (2) selection pressures among protein-coding genes. Phylogenetic reconstruction using concatenation-based methods under partitioned schemes showed congruent topologies between maximum likelihood analysis and Bayesian inference at both family and superfamily levels: Asiloidea and Nemestrinidae may share a closer phylogenetic affinity, while Empidoidea likely occupied a basal position. In this study, we integrate existing mitochondrial genome data and employ phylogenetic analyses to provide new molecular insights for revising the classification system of Asilomorpha.

## 2. Methods

### 2.1. Taxa Sampled and DNA Extraction

Two Asilidae species, *C. jiangxiensis* and *M. xizangensis*, were collected from distinct biogeographic regions: Jing’an County (Jiangxi Province, China; 28°97′ N, 115°26′ E) in August 2022 and Bome County (Xizang Autonomous Region, China; 29°51′ N, 95°46′ E) in July 2023, respectively. Voucher specimens were deposited in 95% ethanol at −20 °C in the Laboratory of Biodiversification of Shaanxi Normal University. Species identification was conducted by Dr. Hao Tang based on morphological diagnostics.

Genomic DNA was extracted from femoral muscle tissue using the DNeasy Blood & Tissue Kit (QIAGEN Group, Beckman Instruments, Inc., Hilden, Germany). To ensure complete cell lysis, tissue was digested in proteinase K buffer at 56 °C for 12 h with continuous agitation at 600 rpm. Subsequent purification followed the manufacturer’s protocol. DNA concentration and purity were quantified using the Qubit 4 Fluorometer with the dsDNA High-Sensitivity assay kit (Invitrogen, Carlsbad, MA, USA). Purified DNA aliquots were cryopreserved at −20 °C in TE buffer.

### 2.2. Data Collection

We determined two new mitochondrial genomes through high-throughput sequencing conducted at Novogene Co., Ltd. (Beijing, China). Specifically, genomic DNA (>200 ng) was used to construct paired-end libraries (350 bp insert size) with the NEB Next^®^ Ultra™ DNA Library Prep Kit (New England Biolabs, Ipswich, MA, USA), followed by sequencing on an Illumina NovaSeq 6000 (Illumina, San Diego, CA, USA) platform (2 × 150 bp reads). Raw reads were quality-filtered using FASTQ v0.19.7 [21] and assembled de novo with MitoZ v2.4 [22] under the invertebrate mitochondrial genetic code. Genome annotation involved the verification of protein-coding genes boundaries through comparative analysis with related taxa in Geneious v11.1.2 [23] following established criteria [24], while tRNA genes were predicted and structurally validated using the MITOS WebServer (http://mitos.bioinf.uni-leipzig.de/index.py, accessed on 7 January 2025). The mitochondrial genomes of *C. jiangxiensis* and *M. xizangensis* were visualized using the *circlize* v0.4.10 [25] package in R.

Additional mitochondrial genomes were obtained from the NCBI database (accessed 31 October 2024), comprising 72 ingroup taxa (representing Asiloidea, Empidoidea, and Nemestrinoidea) and 4 outgroup taxa (Stratiomyomorpha) (Table S1). The data retrieval and filtering procedures were conducted as follows: First, the NCBI database was queried using the keyword “Asilomorpha”, with the genetic compartment restricted to “Mitochondrion” and sequence lengths between 10,000 bp and 30,000 bp. This initial search yielded 2912 records. After removing entries with duplicate species names, 1346 records remained. Further refinement was applied to retain only one representative species per genus, resulting in 547 records. Taxonomic classifications (infraorder, superfamily, and family) for all species were obtained using automated data retrieval scripts. Following established phylogenetic frameworks [11], we retained taxa closely related to Asilidae, primarily distributed across the three superfamilies—Asiloidea, Empidoidea, and Nemestrinoidea. For outgroups, four species from Stratiomyomorpha were randomly chosen based on the criteria outlined by Song et al. [26]. The corresponding mitochondrial genomes were then batch-downloaded from NCBI by their accession numbers. Among the 72 mitochondrial genomes, 29 lacked functional annotations. These sequences were annotated using MitoZ v2.4 [22], followed by manual verification to ensure the completeness of protein-coding genes.

The alignment sequences were obtained from the mitochondrial genomes. Thirteen protein-coding genes were individually extracted as single-gene sequences using Geneious v11.1.2 [23]. Due to the conservation of reading frames, amino acid-level alignments were performed with the invertebrate mitochondrial genetic code implemented in MAFFT v7.526 [27], followed by back-translation to nucleotide sequences. After removing stop codons, the aligned protein-coding genes were concatenated into a supermatrix dataset comprising 11,754 aligned base pairs. This dataset was derived from 13 protein-coding genes across 78 mitochondrial genomes (Table S1).

### 2.3. Gene and Codon Analysis

The Relative Synonymous Codon Usage (RSCU) values for two novel mitochondrial protein-coding genes were computed using CodonW v1.4.2 [28]. RSCU is calculated as the ratio of a codon’s observed frequency to its expected frequency under equal usage of all synonymous codons for a given amino acid [28]. This metric quantitatively assesses codon usage bias, where RSCU = 1 indicates neutral codon usage (no bias), RSCU > 1 represents preferentially used codons, and RSCU < 1 indicates underrepresented codons. The RSCU values were plotted and visualized using the ggplot2 v3.5.2 [29] package in R.

Using the aligned sequences, we computed nonsynonymous (Ka) and synonymous (Ks) substitution rates, along with their ratios (Ka/Ks), for all protein-coding genes using KaKs_Calculator v3.0 [30]. The analysis employed the YN method under the Invertebrate Mitochondrial Genetic Code. The Ka/Ks ratio serves as a measure of selective pressure, where Ka/Ks > 1 indicates positive selection (adaptive evolution), Ka/Ks < 1 reflects purifying selection (functional constraint), and Ka/Ks ≈ 1 suggests neutral evolution. The Ka/Ks ratios were visualized by the ggplot2 v3.5.2 [29] package in R.

### 2.4. Phylogeny Estimation

We reconstructed the maximum likelihood tree from a mitochondrial supermatrix dataset using IQ-TREE v2.1.2 [31] with a partitioned analysis scheme. Protein-coding genes were treated as separate partitions to account for functional divergence, with further partitioning by codon positions to accommodate differential evolutionary rates. The optimal substitution models for each partition were automatically selected using ModelFinder [32] under the Akaike Information Criterion (AIC) with the MERGE option enabled (-m MFP + MERGE) to determine the best partitioning scheme, following an approach analogous to PartitionFinder2 [33]. Tree inference incorporated 1000 ultrafast bootstrap replicates (-UFBoot 1000) for nodal support values while maintaining edge-proportional branch lengths (-p) and allowing partition-specific substitution rates. The resulting phylogeny was visualized using FigTree v1.4.3.

The mitochondrial supermatrix dataset was analyzed using Bayesian inference implemented in MrBayes v3.2.7 [34]. To accommodate heterogeneous evolutionary patterns among protein-coding genes, we determined the optimal data partitioning scheme and nucleotide substitution models using PartitionFinder v2.1.1 [33] under the Bayesian Information Criterion (BIC). The analysis was conducted with the following parameters: two independent MCMC runs with 100 million generations each, sampling every 500 generations, using default prior distributions. Convergence was assessed using Tracer v1.7 [35], ensuring all parameters reached effective sample sizes (ESS) > 200, with the first 25% of samples discarded as burn-in. A maximum clade credibility tree was subsequently generated using TreeAnnotator v2.6.3 [36]. The phylogenetic tree was plotted by the ggtree v3.16.0 [37] package in R.

## 3. Results

### 3.1. Basic Features of Mitochondrial Genomes

**Figure 1:**
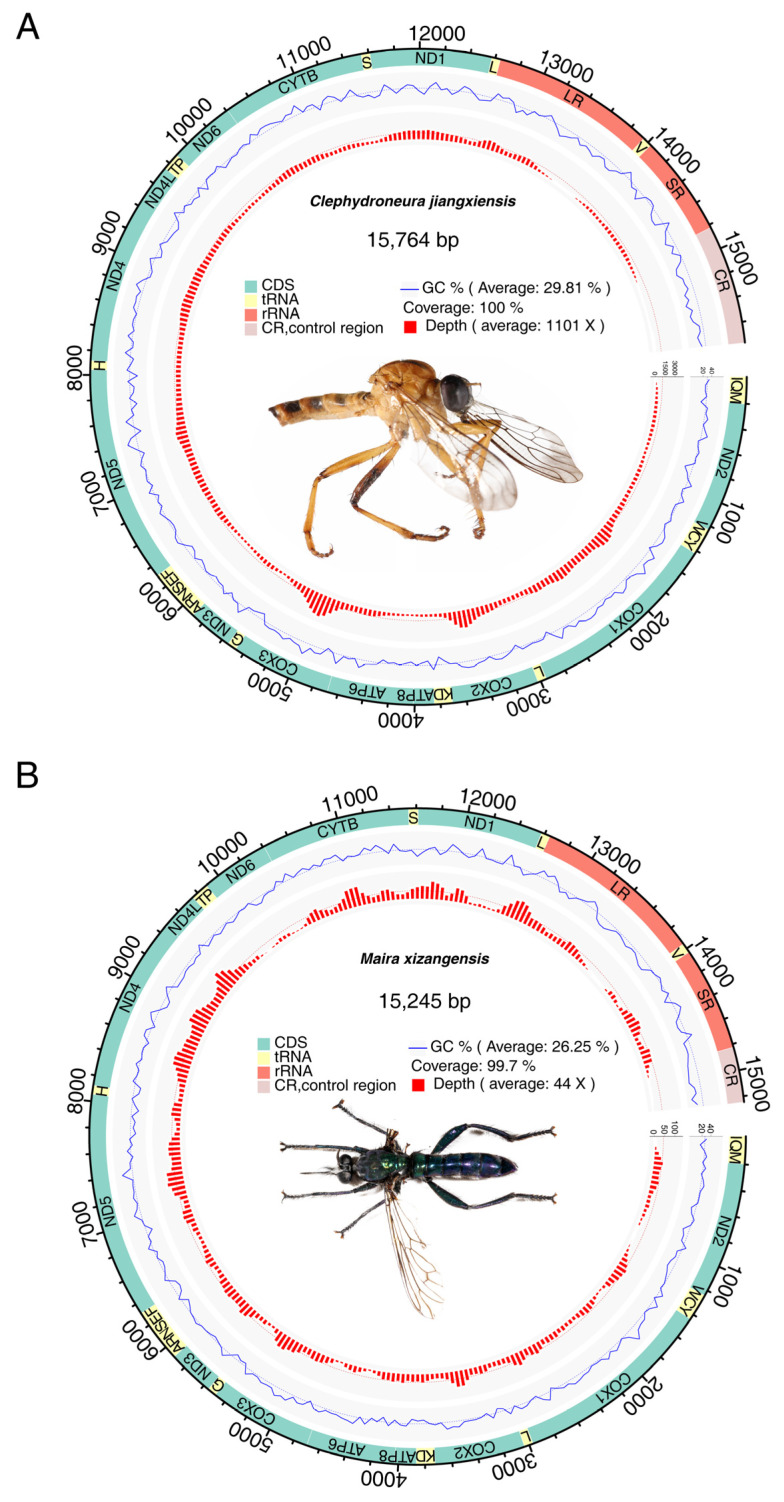
Basic structure of mitochondrial genomes of *C. jiangxiensis* (**A**) and *M. xizangensis* (**B**). The outer circle represents the mitochondrial genome, and the different colors on the circle represent different regions of the mitochondrial genome. Green represents protein-coding genes, red represents rRNA genes, yellow represents tRNA genes, and pink indicates the control region. The blue line in the circle represents the GC content. The red bar charts within the circle represent the sequencing depth, and each bar chart is an average value calculated in units of 50 bases. The images of the insects in the circle were photographed by our team. These two specimens exhibit significant color variation.

We sequenced and annotated the complete mitochondrial genomes of two Asilidae species: *C. jiangxiensis* (15,764 bp) and *M. xizangensis* (15,245 bp) (Figure 1 and Table 1). Both genomes exhibited strong AT bias, with overall AT contents of 70.2% and 73.8%, respectively. Detailed nucleotide composition analysis showed the following base frequencies: A = 41.5%; C = 19.5%; G = 10.3%; T = 28.7% (*C. jiangxiensis*); A = 39.2%; C = 16.2%; G = 10.1%; T = 34.6% (*M. xizangensis*). The control region accounted for 6.0% (943 bp) and 2.8% (422 bp) of the respective genomes. The comparative analysis of coding sequences revealed length polymorphisms and species-specific AT content patterns (Table S2). Both mitochondrial genomes contained the standard 37 metazoan mitochondrial genes (13 protein-coding genes, 22 tRNAs, and 2 rRNAs), all exhibiting the ancestral insect gene arrangement first characterized by Clary and Wolstenholme [38]. The complete conservation of this gene order without any rearrangements underscores the evolutionary stability of mitochondrial architecture in these dipteran lineages.

**Figure 2:**
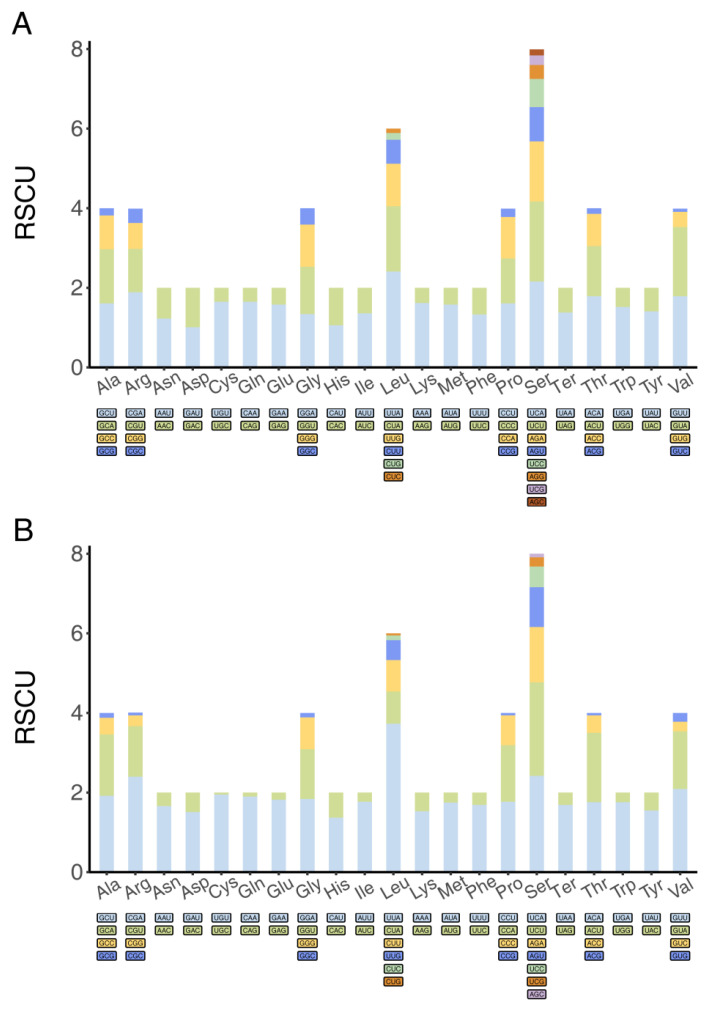
Relative synonymous codon usage (RSCU) of mitochondrial protein-coding genes. (**A**) *C. jiangxiensis*; (**B**) *M. xizangensis*. In the stacked bar plot, each colored segment represents the usage frequency of a specific codon.

The analysis of mitochondrial protein-coding genes in *C. jiangxiensis* and *M. xizangensis* revealed conserved codon usage patterns characteristic of insect mitochondrial genomes (Figure 2). Serine (Ser) and leucine (Leu) emerged as the most abundant amino acids, primarily encoded by TCA (Ser) and TTA (Leu), respectively, consistent with findings in other insect mitochondrial genomes [39,40]. RSCU analysis demonstrated a pronounced preference for A/U-ending codons, with 100% of preferred codons terminating in either adenine or uracil (Figure 2B). This bias likely reflects translational selection pressure, as A/U-rich codons may enhance translational efficiency through improved tRNA-mRNA pairing in the mitochondrial system [41]. The observed patterns further support the hypothesis that mitochondrial genetic codes are under strong selective constraints to maintain optimal translation rates in these dipteran species.

We evaluated evolutionary rate variation across codon positions through substitution saturation analysis of protein-coding genes using CodonW v1.4.2. The results demonstrated significant positional heterogeneity (Table S3): first and second codon positions showed limited saturation (Iss values significantly below Iss.cSym), indicating strong purifying selection [42]; third positions exhibited substantial saturation (Iss = 0.79 ± 0.05), exceeding the critical threshold (Iss.cSym = 0.809) at our sampling depth of 32 OTUs. This differential saturation pattern reflects the well-documented phenomenon of elevated evolutionary rates at synonymous third codon positions compared to functionally constrained first and second positions. The results suggest that while amino acid-altering substitutions face strong selective constraints, synonymous sites accumulate mutations more freely, which is consistent with neutral evolutionary expectations for mitochondrial protein-coding genes.

**Figure 3:**
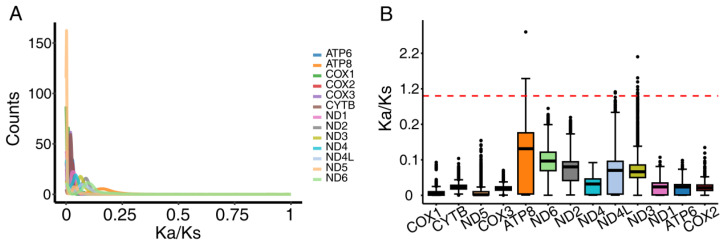
The nonsynonymous/synonymous (Ka/Ks) ratio analysis of 13 mitochondrial protein-coding genes among 78 taxa. (**A**) Distribution of Ka/Ks ratios of all genes. In the picture, each color represents a gene. (**B**) Differences in Ka/Ks ratios of 13 protein-coding genes. In the box plot, each gene is represented by a different color. The solid black dots in the figure represent outliers. The red horizontal line means that Ka/Ks is equal to 1. For ordinates, when the Ka/Ks ratio is greater than 0.2, the value is transformed by (kaks − 0.2)/10 + 0.2.

The nonsynonymous-to-synonymous substitution rate ratio (ω = Ka/Ks) revealed distinct selective pressures across mitochondrial protein-coding genes (Figure 3). Core respiratory components (ATP6, COX1-3, CYTB, ND1-2, and ND4-6) exhibited strong purifying selection (mean ω = 0.03 ± 0.04), with COX1 showing the strongest constraint (ω = 0.01 ± 0.01), consistently with its critical role in electron transport chain stability. In contrast, accessory proton channel genes (ATP8, ND3, and ND4L) demonstrated relatively relaxed selection (ω = 0.11 ± 0.12 for ATP8), with 0.04% of species pairs (16/40,000 comparisons) showing potential positive selection (ω > 1). This evolutionary pattern reflects (1) stringent structural/functional constraints on core oxidative phosphorylation machinery, and (2) greater tolerance for variation in proton channel regulation components. The differential selection pressures correlate strongly with each gene’s functional importance in mitochondrial bioenergetics.

### 3.2. Phylogenetic Analysis

**Figure 4:**
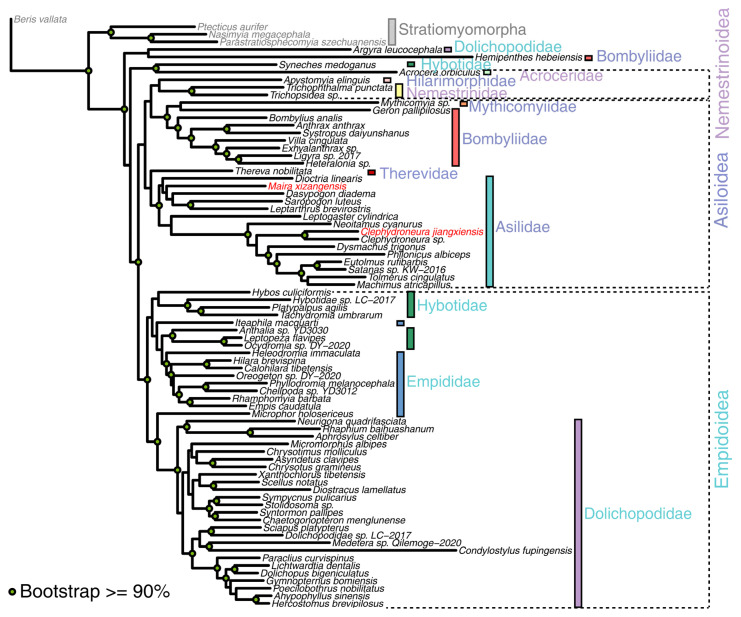
The maximum likelihood tree inferred from the mitochondrial supermatrix dataset of 78 taxa. In the phylogeny, nodes with strong statistical support (bootstrap values > 90%) are indicated by solid green circles. The newly sequenced mitochondrial genomes are marked in red. Outgroups are labeled in gray. Taxonomic information was obtained from the NCBI Taxonomy Database. Families (colored bars) and superfamilies (font color) are visually distinguished.

Phylogenetic reconstruction based on a supermatrix dataset comprising 78 species yielded a maximum likelihood tree with strong nodal support (64% bootstrap values > 90%) (Figure 4). The analysis robustly supported the monophyly of Asilidae (bootstrap value = 100%), with *C. jiangxiensis* and *M. xizangensis* firmly placed within this clade. However, the monophyly of Asiloidea was not recovered in our topology. In this tree, we identified a clade comprising Asilidae + Therevidae, which formed a closer relationship to the combined lineage of Bombyliidae, Mythicomyiidae, Nemestrinidae, and Hilarimorphidae. Notably, Nemestrinidae clustered according to previous research within Nemestrinoidea rather than Asiloidea. Another branch also revealed complex relationships within the Empidoidea superfamily. The analysis identified a paraphyletic group comprising Hybotidae and Empididae, with Dolichopodidae positioned as their sister lineage. Notably, two nominal Empidoidea species—*H. hebeiensis* (Bombyliidae) and *Argyra leucocephala* (Dolichopodidae)—were unexpectedly placed at the basal position of the tree (bootstrap value = 82%). Another branch comprising *Syneches medoganus* (Empidoidea: Hybotidae) and *Acrocera orbiculus* (Nemestrinoidea: Acroceridae) also had high support (bootstrap value = 97%), but they were separated from Empidoidea and Nemestrinoidea.

**Figure 5:**
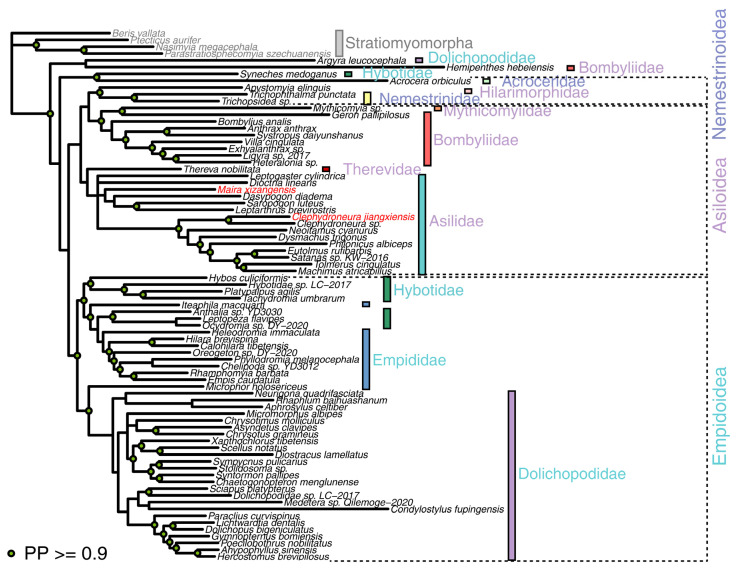
The Bayesian analysis of the mitochondrial supermatrix dataset of 78 taxa. In the phylogeny, nodes with high posterior probability (PP > 0.9) are indicated by solid green circles. The newly sequenced mitochondrial genomes are marked in red. Outgroups are labeled in gray. Taxonomic information was obtained from the NCBI Taxonomy Database. Families (colored bars) and superfamilies (font color) are visually distinguished.

Bayesian analysis based on a supermatrix dataset comprising 78 species yielded a phylogenetic tree with high posterior probability (67% posterior probability > 0.9) (Figure 5). In this phylogenetic tree, *C. jiangxiensis* and *M. xizangensis* were placed within Asilidae, with Asilidae being recovered as a monophyletic group. Moreover, the phylogenetic relationships of other families and superfamilies were consistent with the results from the maximum likelihood analysis. Only a few species exhibited inconsistent phylogenetic placements within their respective families compared to the maximum likelihood tree, such as *Neoitamus cyanurus*.

## 4. Discussion

Mitochondrial genomes have been extensively employed in insect phylogenetics due to their structural simplicity, strict maternal inheritance, and relatively high evolutionary rates, which collectively provide resolution across broad taxonomic scales [24]. In this study, we performed phylogenetic analyses of 74 published dipteran mitochondrial genomes using a concatenation-based method to elucidate systematic relationships among three superfamilies. Both maximum likelihood analysis and Bayesian inference yielded consistent topologies at family and superfamily levels. While minor inconsistencies were observed in the placement of some terminal taxa (e.g., *N. cyanurus*), these did not affect higher-level phylogenetic interpretations. For brevity, we focus the subsequent discussion on the maximum likelihood results as a representative phylogenetic framework.

Our phylogenetic analyses of mitochondrial protein-coding genes in three dipteran superfamilies (Asiloidea, Empidoidea, and Nemestrinoidea) identified both consistencies and conflicts with existing taxonomic classifications. The maximum likelihood tree strongly supported the monophyly of Asilidae. However, multiple traditionally recognized families exhibited paraphyletic or polyphyletic relationships, particularly among Bombyliidae and Hybotidae lineages. These results indicated that current classification might be affected ambiguous taxonomic definitions or insufficient sampling coverage. Our phylogenetic framework enables a critical re-examination of the systematic controversies surrounding these three groups.

Although the concept of Asiloidea was formalized in the mid-20th century [43], the question of their monophyly had been debated, particularly regarding the inclusion of Bombyliidae. Previous studies consistently supported the monophyly of Asiloidea only when Bombyliidae were excluded [11,13,16]. In our maximum likelihood tree, Bombyliidae were placed as a sister family to Asilidae + Therevidae, reflecting the close relationship observed in other phylogenetic studies [13]. However, the phylogenetic position of *H. hebeiensis* (Bombyliidae) questioned the stability of Bombyliidae’s relationship with other Asiloidea members. Furthermore, while the previous classification system included Hilarimorphidae within Asiloidea, our phylogenetic tree showed this family to be more distantly related to the Asilidae + Therevidae. This observation aligns with Shin’s [16] proposal that Hilarimorphidae should be a sister family to Homeodactyla. Additionally, although Mythicomyiidae were classified within Asiloidea and appear closely related to Bombyliidae in our maximum likelihood tree, this relationship received negligible support.

Another key unresolved question concerns the phylogenetic relationships within the Nemestrinoidea superfamily and its systematic position. Historically, Nemestrinoidea were recognized as comprising two families, Nemestrinidae and Acroceridae [44,45]—a sister-group relationship subsequently supported by molecular phylogenetic analyses [16]. However, our maximum likelihood tree revealed that these families occupy distinct branches, suggesting considerable phylogenetic divergence. This result aligned with longstanding controversies regarding their relationships, as evidenced by conflicting morphological and molecular datasets [11,13,46,47,48]. Consequently, the precise phylogenetic placement of Nemestrinoidea remains problematic to determine.

The potential sister-group relationship between Empidoidea and Asiloidea represents the third significant phylogenetic question. While morphological evidence supported their sister-group status [44,45], molecular studies had not explicitly addressed this relationship. Instead, molecular data consistently recovered a sister-group relationship between Asiloidea and Eremoneura (containing Empidoidea and Cyclorrhapha) [13,16,26]. Our maximum likelihood tree based on mitochondrial genomes showed Empidoidea and Asiloidea as sister branches, while two taxa (*S. medoganus* and *A. leucocephala*) appear at the phylogenetic tree’s base. This inconsistency suggests that mitochondrial genomic data alone remain insufficient for conclusively determining the phylogenetic relationship between Empidoidea and Asiloidea, highlighting the need for broader genomic sampling.

In this study, we reconstructed the phylogeny of Asilomorpha using mitochondrial genomes, revealing unresolved phylogenetic conflicts among three groups (Asiloidea, Empidoidea, and Nemestrinoidea). These conflicts may arise from the following: (1) Limitations of single-marker analysis. While mitochondrial genomes are widely used for preliminary phylogenetic studies, reliance solely on this marker may lead to biased inferences for rapidly radiating groups, particularly due to incomplete lineage sorting (ILS). Integrating multi-locus genomic data is therefore essential to improve resolution, e.g., SNPs and UCEs. (2) Insufficient taxonomic sampling. The current dataset contains limited representatives from the three superfamilies. Such sparse sampling may obscure true phylogenetic signals. Future studies should include broader taxonomic coverage at the family level to elucidate Asilomorpha’s evolutionary history. (3) Need for morphological validation. Controversial taxa require the re-examination of morphological synapomorphies to corroborate molecular phylogenetic hypotheses.

## 5. Conclusions

In this study, we sequenced the complete mitochondrial genomes of two Chinese endemic species, *C. jiangxiensis* and *M. xizangensis*, and characterized their structural organization and codon usage patterns. These new sequences fill a gap in the mitochondrial genomic database of Asilidae and will facilitate future studies on genus-level phylogeny within this family.

Combined with published mitochondrial genomes from three superfamilies, we reconstructed phylogenetic trees. Our analyses suggest that Asiloidea and Nemestrinidae (Nemestrinoidea) are placed in a clade, likely as sister groups. However, the basal position of Acroceridae (also within Nemestrinoidea) obscures the exact relationship between Asiloidea and Nemestrinoidea. Although Empidoidea were recovered as a sister lineage to Asiloidea + Nemestrinidae in both analyses, the aberrant placement of *H. hebeiensis* cast doubt on this hypothesis. While our mitochondrial genome-based study provides novel insights into the phylogeny of these groups, resolving their evolutionary history conclusively will require additional data from nuclear genomes and broader taxon sampling. In future studies, we will continue to collect specimens from all families within the three superfamilies, with particular emphasis on the Asilidae group. For the collected specimens, in addition to obtaining mitochondrial genome data, we will also gather single-nucleotide polymorphism (SNP) data.

## Figures and Tables

**Table 1:** The structural characteristics of the mitochondrial genomes of *C. jiangxiensis* and *M. xizangensis*.

Feature	A (%)	C (%)	G (%)	T (%)	AT (%)	Size (bp)	Proportion in the Genome (%)
*C. jiangxiensis*							
genome	41.5	19.5	10.3	28.7	70.2	15,764	100
PCGs	40.3	21.5	11.3	26.9	67.2	11,212	71.1
rRNA	45.2	16.6	7.6	30.7	75.9	2084	13.2
tRNA	40.9	14.7	11.1	33.2	74.1	1473	9.3
Control region	47.1	10.2	4.3	38.4	85.5	943	6.0
*M. xizangensis*							
genome	39.2	16.2	10.1	34.6	73.8	15,245	100
PCGs	38.0	17.2	10.7	34.1	72.1	11,230	73.7
rRNA	43.4	14.8	8.0	33.8	77.2	2104	13.8
tRNA	39.4	13.7	10.7	36.3	75.7	1463	9.6
Control region	47.9	5.7	1.7	44.8	92.7	422	2.8

## Data Availability

The mitochondrial genome data are publicly available from the National Center for Biotechnology Information.

## Supplementary Materials

The following supporting information can be downloaded at: https://www.mdpi.com/article/10.3390/genes16050561/s1, Table S1. Taxonomic information and Genbank accession number for 78 taxa. Table S2. Basic information of mitochondrial encoded genes of *C. jiangxiensis* and *M. xizangensis*. Table S3. Substitutions saturation analysis in different codons of 78 mitochondrial protein-coding genes.
